# Telocyte dynamics in psoriasis

**DOI:** 10.1111/jcmm.12601

**Published:** 2015-05-19

**Authors:** CG Manole, Mihaela Gherghiceanu, Olga Simionescu

**Affiliations:** a Department of Cell Biology and Histology, ‘Carol Davila’ University of Medicine and PharmacyBucharest, Romania; b Laboratory of Ultrastructural Research, ‘Victor Babeş’ National Institute of PathologyBucharest, Romania; c Department of Dermatology, Colentina University Hospital, ‘Carol Davila’ University of Medicine and PharmacyBucharest, Romania

**Keywords:** Telocytes, psoriasis, Langerhans cells, dendritic cells, papillary dermis, angiogenesis, Auspitz’s sign

## Abstract

The presence of telocytes (TCs) as distinct interstitial cells was previously documented in human dermis. TCs are interstitial cells completely different than dermal fibroblasts. TCs are interconnected in normal dermis in a 3D network and may be involved in skin homeostasis, remodelling, regeneration and repair. The number, distribution and ultrastructure of TCs were recently shown to be affected in systemic scleroderma. Psoriasis is a common inflammatory skin condition (estimated to affect about 0.1–11.8% of population), a keratinization disorder on a genetic background. In psoriasis, the dermis contribution to pathogenesis is frequently eclipsed by remarkable epidermal phenomena. Because of the particular distribution of TCs around blood vessels, we have investigated TCs in the dermis of patients with psoriasis vulgaris using immunohistochemistry (IHC), immunofluorescence (IF), and transmission electron microscopy (TEM). IHC and IF revealed that CD34/PDGFRα-positive TCs are present in human papillary dermis. More TCs were present in the dermis of uninvolved skin and treated skin than in psoriatic dermis. In uninvolved skin, TEM revealed TCs with typical ultrastructural features being involved in a 3D interstitial network in close vicinity to blood vessels in contact with immunoreactive cells in normal and treated skin. In contrast, the number of TCs was significantly decreased in psoriatic plaque. The remaining TCs demonstrated multiple degenerative features: apoptosis, membrane disintegration, cytoplasm fragmentation and nuclear extrusion. We also found changes in the phenotype of vascular smooth muscle cells in small blood vessels that lost the protective envelope formed by TCs. Therefore, impaired TCs could be a ‘missed’ trigger for the characteristic vascular pathology in psoriasis. Our data explain the mechanism of Auspitz’s sign, the most pathognomonic clinical sign of psoriasis vulgaris. This study offers new insights on the cellularity of psoriatic lesions and we suggest that TCs should be considered new cellular targets in forthcoming therapies.

## Introduction

The cellularity of the dermis is perceived to be comprised of fibroblasts, endothelial cells, pericytes, dendritic cells (DCs), immune cells, macrophages, nerve endings, smooth muscle cells and the recently described telocytes (TCs) 1–3. TCs are not specific to the dermis (for more details, see www.telocytes.com) and have been described in the interstitium of many organs 4–24. TCs are characterized by the presence of very long and slender moniliform cellular prolongations termed telopodes (Tps). The thickness of the thin segments of Tps (podomers) is comparable to that of collagen fibrils. The podoms (dilated segments) accommodate mitochondria, endoplasmic reticulum, and caveolae 2,3,25. Recently, the most advanced 3D microscopy technique (FIB-SEM tomography) revealed the spatial conformation of human dermal TCs and their Tps and extracellular vesicles 26. In human skin, TCs are key components of stem cells niches, where they physically interact with stem cells and other interstitial cells, suggesting an unexplored potential of TCs in skin regeneration and repair 2. Many studies have showed that TCs are completely different from fibroblasts in terms of cell culture 27,28, ultrastructure 3,24,29,30, miRNA imprint 31, gene profile 32–34 and proteomics 35.

The involvement of TCs in skin pathology has been shown in scleroderma patients; TCs are numerically reduced in their skin and exhibit numerous ultrastructural particularities, from increased cell volume in the early stage to hallmarks of cellular degeneration in later stages 36–38. The involvement of TCs in other pathologies has also been reported 39.

DCs are cellular participants in the chronic skin inflammatory process that characterizes psoriasis 40–45. Four subtypes of DCs are known: Langerhans cells (LCs), dermal dendritic cells (DDCs), inflammatory dendritic epidermal cells (iDCs) and plasmacytoid dendritic cells (pDCs); though their specific role(s) are unclear, a notably increased number suggests their involvement in the psoriasis adaptive immune response: 46–49. Currently, LCs are the most studied type of DC, and their phenotype has been extensively described by immunohistochemical and ultrastructural analysis 50–54. The rest of the DC subtypes have been immunohistochemically characterized: (*i*) immature DDCs express CD11c and mature DDCs express CD83 and CD208 (dendritic cells lysosomal associated membrane protein, DC-LAMP) 55; (*ii*) iDCs express CD11c, CD14, CD209, nitric oxide synthase (NOS) 43,56; and (*iii*) pDCs express CD11c, CD123, CD205 and TNFα 57–59.

The ability of TCs to establish cellular contacts (either physical or paracrine) with immune cells has been documented in other organs, including skin 2,3,25,29,36,60–63. Thus, in the context of the vast immunology of psoriasis, it is tempting to presume that TCs could be involved in disease initiation and/or progression. Furthermore, (neo-)angiogenesis is at least partially responsible for the clinical signs of psoriasis 64–68. Previous studies have shown that, within the intense metabolic border zone of myocardial infarction lesions, TCs are involved in neo-angiogenesis, proving their involvement in the reparatory process 69. Therefore, the involvement of TCs in angiogenesis in psoriasis should be investigated.

In this study we investigated the presence, density and distribution of TCs as a distinct interstitial cell population in the dermis of psoriasis patients. We also assessed whether psoriatic skin TCs exhibit (ultra)structural changes. TCs distribution and the pattern of cellular interaction in psoriasis patients could offer new insights into the pathogenesis and progression of this disease.

## Material and methods

### Patients

We studied skin samples from 10 patients (5 males and 5 females) with fully developed (mature) plaques of psoriasis vulgaris. Three of the patients were diagnosed with psoriasis vulgaris type II, and seven were diagnosed with psoriasis vulgaris type I. The triggers of psoriasis were psychological (*n* = 8) or metabolic (*n* = 2). The skin samples were biopsied three times: from the mature psoriatic lesion, non-lesional skin (40 cm distance from any lesion), and after the clearance induced by local treatment. The treatment followed by the patients was entirely topical: keratolytic (urea and salicylic acid) and cytoreductor (based on Anthraline), followed by calcipotriol and steroid ointments. This study was approved by the Bioethics Committee of the ‘Carol Davila’ University of Medicine and Pharmacy, Bucharest, according to generally accepted international standards. All subjects provided signed informed consent.

### Biopsies

After taking a clinical history and explaining the procedure to the patient, they undressed and the sites of biopsy were chosen. Each of the areas was prepared with a betadine swab to insure sterile conditions. The area was then injected subepidermally with lidocaine HCL 1% (Xilina, Sicomed, Bucharest, Romania) using a 1 ml syringe until a bleb approximately 5 mm diameter formed under the skin. After testing for numbness, the biopsy was performed with a sterile 6 mm skin punch. After the skin was cored and excess blood cleared the fragment of skin, the biopsy was removed using a scalpel and forceps. The post-biopsy lesion was ligated with two stitches of Ethicon Polyglactin 910 (Somerville, NJ, USA). To prevent infection, the wound was dressed with bacitracin Zn and neomycin sulphate powder (Baneocin, Sandoz, Austria) and carefully bandaged.

Each of the skin biopsies were divided into two equal fragments, each fragment following the protocol for paraffin embedding for histology, immunohistochemistry (IHC) and immunofluorescence (IF) or Epon embedding for transmission electron microscopy (TEM).

### Histology and immunohistochemistry

Histology and IHC were performed on formalin-fixed, paraffin-embedded, 3-μm-thick tissue sections made from the tissue samples collected from all patients. For histology, standard haematoxylin and eosin staining was performed. For IHC, samples were incubated with primary antibodies (Table1) overnight according to standard protocol 20. We used the Novolink™ Max Polymer Detection System (Leica, New Castle Upon Tyne, UK). Counterstaining was done with haematoxylin, chloral-hydrate and lithium carbonate. Images were acquired using a (CCD) Axiocam HRc Zeiss camera with AxioVision software (Carl Zeiss Imaging solution GmbH, Oberkochen, Germany) on a Nikon Eclipse E600 microscope (Nikon Instruments Inc., Tokyo, Japan).

**Table 1:** The primary antibodies used for the IHC

Antibody	Cell	Dilution	Clone	Producer
CD31	Endothelial cells	1:50	mJC70A	Dako
CD34	Telocytes	1:50	mQBEnd10	Dako
CD117	Melanocytes	1:200	mYR145	Cell Marque, Rocklin, CA, USA
col4	Basal membrane	1:100	mPHM-12	Leica
PDGFRa	Telocytes	1:40	Polyclonal	Neomarkers
S100	Langerhans cells	1:400	Polyclonal	Dako

### Immunofluorescence

For IF we used formalin-fixed paraffin-embedded 3-μm-thick tissue sections. After deparaffinization, the samples were buffered at 97°C with Epitope Retrieval Solution (Novocastra, Leica) at pH 6 for PDGFRα and pH 9 for CD34. The samples were washed in PBS with glycine (2 mg/ml) and then blocked with 2% BSA for 1 hr. Samples were incubated with primary antibodies overnight at room temperature with a cocktail consisting of mouse monoclonal CD34 (1:50, clone QBEnd10; Dako, Glostrup, Denmark) and rabbit polyclonal PDGFRα (1:40; Neomarkers, Fremont, CA, USA). After washing three times in EnVisionTM Flex Wash Buffer (Dako) the sections were incubated with anti-mouse Alexa Fluor 488 (1:200; Life Technologies, Molecular Probes, Grand Island, NY, USA) or anti-rabbit rhodamine (1:200; Life Technologies, Molecular Probes) secondary antibodies for another 2 hrs. The nuclei were stained with 4′,6-diamidino-2-phenylindole (DAPI; Life Technologies, Molecular Probes). Immunofluorescence studies were performed with a Zeiss Axio Imager Z1 microscope (Carl Zeiss MicroImaging GmbH) using 10×, 20×, 40×, and 63× objectives with the appropriate fluorescence filters. Digital images were acquired with a CV-M4+CL CCD camera (JAI, Yokohama, Japan) linked to a computer running the Isis software (Metasystems GmbH, Altlussheim, Germany).

### Cell counting

Cell counting was performed on calibrated IF images of known magnification using the NIH ImageJ software 70. Only cells with DAPI-positive nuclei that presented with positive expression for CD34 (green), PDGFRα (red) or both CD34/PDGFRα (yellow) were considered for the analysis. The counted cells were reported for a total surface of 1 mm^2^. The summation of the surfaces of known magnification images was used to obtain this surface area. Data were processed and statistically analysed using Microsoft Excel software.

### Transmission electron microscopy

Transmission electron microscopy was performed on small (1 mm^3^) tissue fragments processed according to a routine Epon embedding procedure as described previously 2. Light microscopy was performed on 1 μm semi-thin sections stained with 1% toluidine blue and digital images recorded using a CCD Axiocam HRc Zeiss camera with AxioVision software (Carl Zeiss Imaging Solution GmbH) on a Nikon Eclipse E600 microscope (Nikon Instruments, Inc.). Thin sections (∽60 nm) were examined with a Morgagni 268 transmission microscope (FEI Company, Eindhoven, the Netherlands) at 80 kV. Digital electron micrographs were acquired with a MegaView III CCD and iTEM-SIS software (Olympus, Soft Imaging System GmbH, Münster, Germany). To highlight the TCs and Tps, TEM images were digitally coloured in blue using Adobe© Photoshop CS3.

## Results

**Figure 1:**
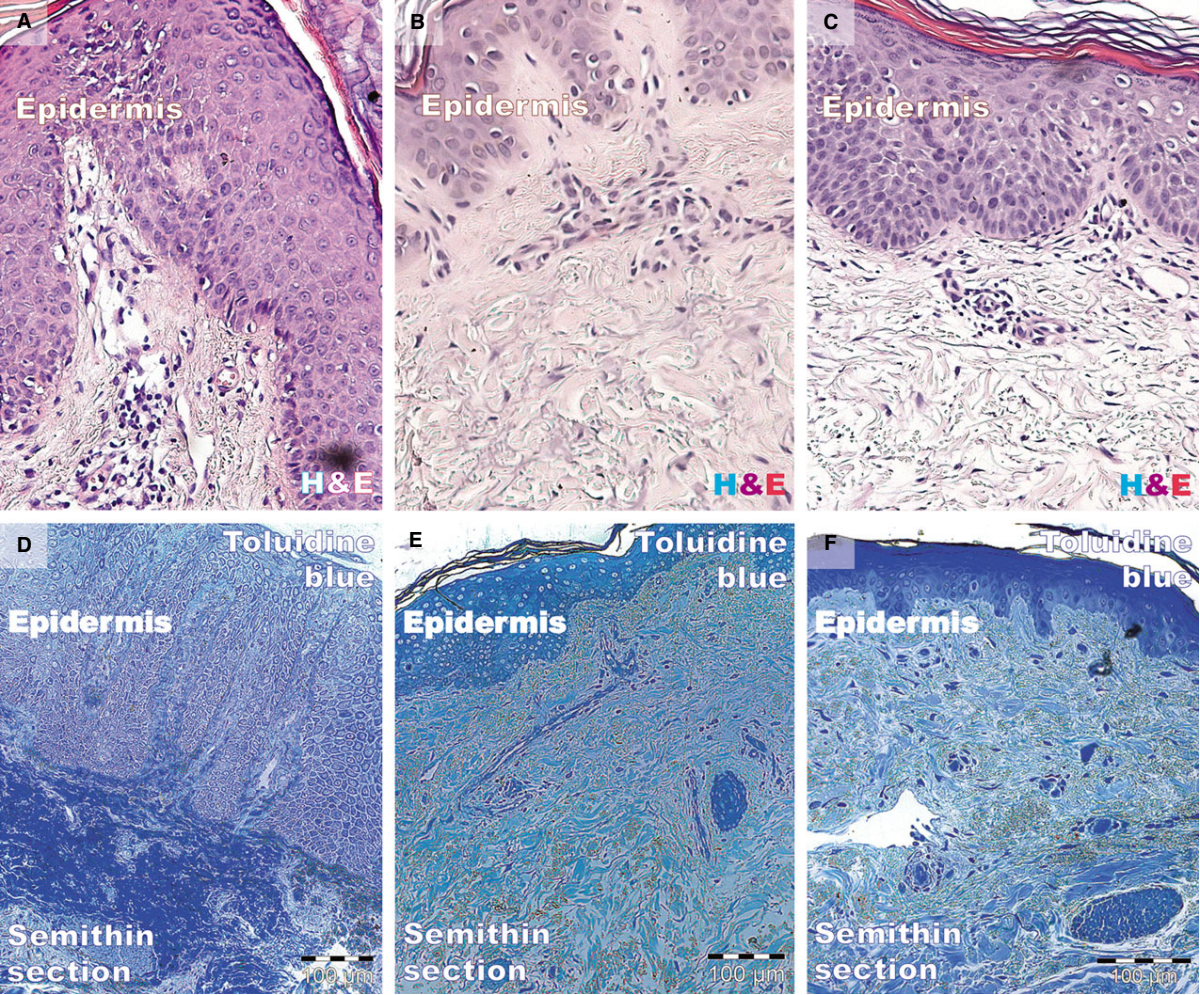
Light microscopy of paraffin-embedded (A–C, haematoxylin and eosin) and resin-embedded (D–F, toluidine blue) skin biopsies. (A and D) The epidermis of a psoriatic plaque exhibits acanthosis, elongation of rete ridges, and hyperkeratosis with parakeratosis (alternating with orthokeratosis). The papillary dermis is oedematous with dilated blood vessels and inflammatory infiltrate (mainly lymphocytes), which is also in the reticular dermis. (B and E) The epidermis, papillary dermis, and reticular dermis have a normal appearance. (C and F) The thickness of the epidermal layer is similar to that of uninvolved skin. Epithelial cells have a normal appearance in accordance with their position in the epidermis. The dermis is still oedematous but with scarce inflammatory infiltrate. Magnification 200×.

Histology using haematoxylin and eosin and toluidine blue staining is shown in Figure1. The biopsies taken from fully developed plaques exhibited the hyper-proliferation aspect of psoriasis: acanthosis (thickening of the epidermis) with characteristic elongation of the rete ridges (tips are clubbed with a tendency to fuse to one another); hyperkeratosis with parakeratosis (alternating with orthokeratosis); and dilated and tortuous blood capillaries in the papillary dermis, with mild oedema, exhibiting chronic inflammatory infiltrate (mainly small lymphocytes). The reticular dermis also contained dilated blood vessels with superficial perivascular infiltration of lymphocytes.

Biopsies from the non-lesional skin showed the normal aspect. Samples from treated patients had decreased epidermal thickness and a smaller rete ridge height. Epithelial cells appeared normal, corresponding to their morphology and staining properties in the epidermal layers. The dermis (papillary or reticular) had decreased inflammatory infiltrate, and the mild oedema was slightly persistent. Eosinophils and neutrophils were absent from the inflammatory infiltrate. In contrast, the distant uninvolved skin and treated lesion had fewer normally maturated keratinocyte layers and similar rete ridge lengths.

**Figure 2:**
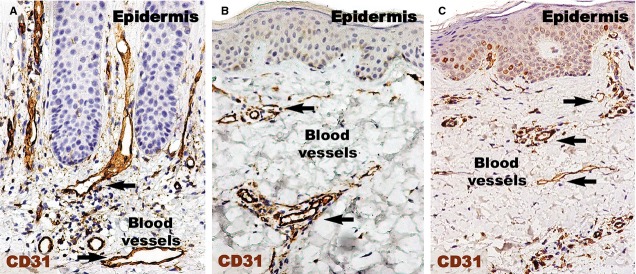
CD31 immunohistochemistry of a psoriatic plaque (A), distant uninvolved skin (B), and treated skin (C). (A) The density of blood vessels (black arrows) and their diameters are greater in the papillary dermis of a psoriatic plaque compared to uninvolved skin (B). (C) Even if slightly denser, the diameters of blood vessels in the treated skin are comparable to those of uninvolved skin from (B). Magnification 200×.

CD31 expressed (Fig.2) in the human dermis was increased in the psoriatic plaque compared to distant uninvolved skin. In the psoriatic plaque, the blood vessels were enlarged and had a sinuous trajectory within the papillary dermis. On the other hand, the cellularity around the vessels and CD31 expression was increased. However, we documented a marked reduction in the expression of CD31 in the treated lesions compared to psoriatic lesions, but still higher than in the uninvolved skin. Within the treated psoriatic plaques, CD31-positive endothelial cells were seen in blood vessels in the tips of the dermal papillae.

**Figure 3:**
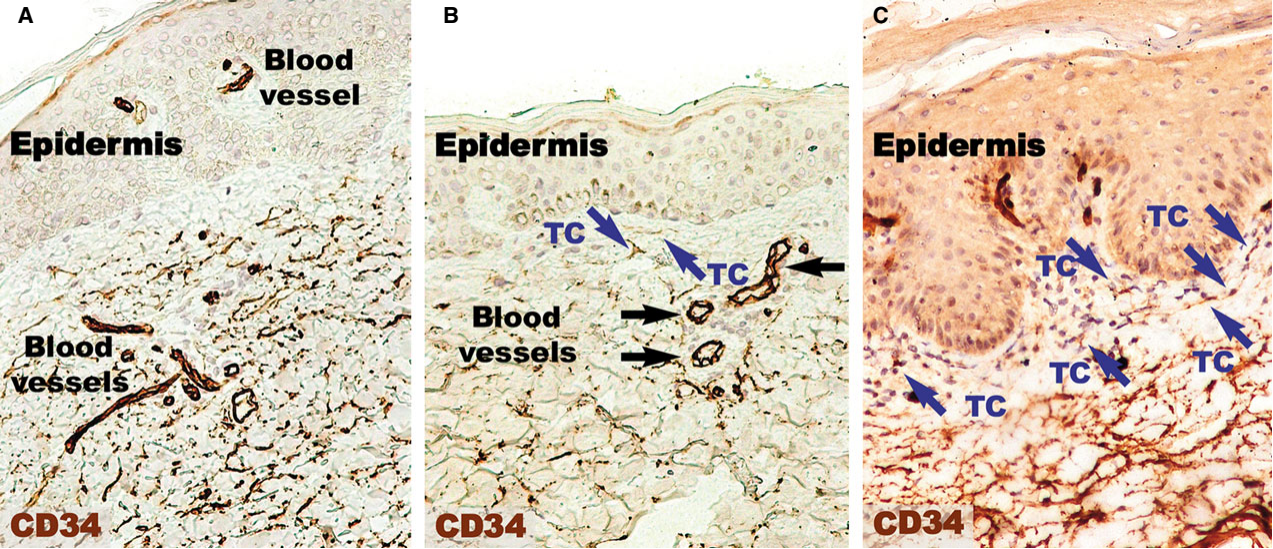
CD34 immunohistochemistry of a psoriatic plaque (A) revealing a lower density of positive expression in the papillary dermis compared to distant uninvolved skin (B). However, the papillary dermis of treated skin has a density comparable to uninvolved skin (C). CD34 expression is higher within the reticular dermis of psoriatic skin (A) and treated skin (C) compared the reticular dermis of normal skin. Silhouettes of CD34-positive telocytes (TC; blue arrows) with long telopodes were observed in the papillary dermis of normal skin (A) and treated skin (C). Magnification 200×. Black arrows indicate blood vessels.

Endothelial cells were also CD34-positive (Fig.3). Compared to CD31 positivity, the positive perivascular expression was higher and apparently uniform in the uninvolved skin. Within uninvolved skin dermis, the density of CD34 expression in cellular branched shapes was higher within the adjacent perivascular territory. In psoriatic plaques, CD34 was uniformly increased in the reticular dermis. The papillary dermis seemed to lack CD34-positive structures.

**Figure 4:**
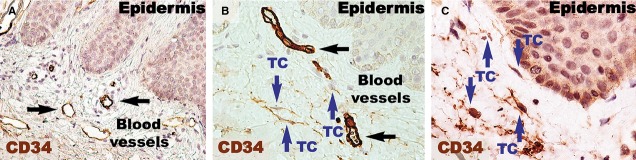
CD34 immunohistochemistry reveals reduced expression in the papillary dermis of a psoriatic plaque (A) compared to the papillary dermis of distant uninvolved skin (B) and treated skin (C). (A) The blood vessels are more numerous and dilated in the psoriatic plaque dermis compared to (B and C). Also, no CD34 positivity was found in interstitial cells in the papillary dermis. (B) In the papillary dermis of uninvolved skin, telocytes (TC; blue arrows) appear positive for CD34 in the vicinity of blood vessels. (C) The presence of CD34-positive telocytes (TC; blue arrows) was also noted in the papillary dermis of treated skin, with telopodes running parallel to the basement membrane of the epidermis. Black arrows indicate blood vessels. Magnification 400×.

Higher magnifications (Fig.4) of distant uninvolved skin revealed CD34-positive cells in a fusiform silhouette with very long cellular prolongations. These interstitial cells are distinct from endothelial cells and specifically located in close proximity of blood vessels within the papillary dermis. Such CD34-positive cells are not present near the dilated capillaries in the dermis of psoriatic plaques. In the treated lesional skin, the general expression of CD34 was slightly increased compared to uninvolved skin. Fusiform cells were present in the papillary dermis, parallel and in close proximity to the basement membrane. Moreover, these cells were situated in the proximity of blood vessels.

**Figure 5:**
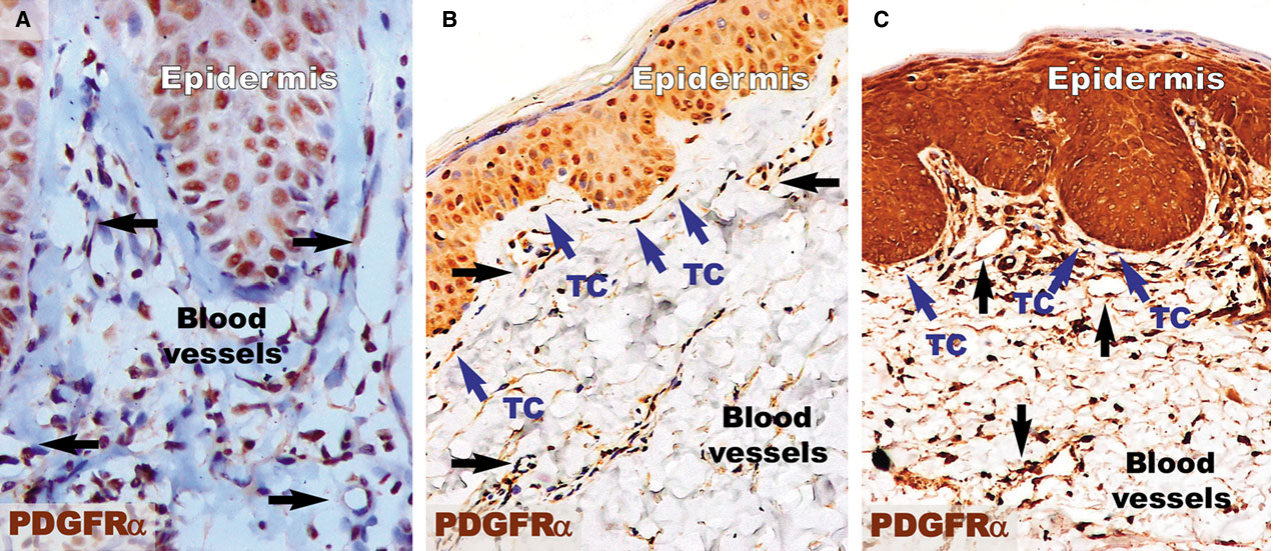
PDGFRα immunohistochemistry revealed an increased density of PDGFRα-positive structures in the papillary dermis of a psoriatic plaque (A) and treated skin (C) compared to the papillary dermis of distant uninvolved skin (B). (A) The papillary dermis of the lesional skin has increased cellularity, but no PDGFRα-positive telocytes were observed. Blood vessels (black arrows) have dilated diameters within the dermis. (B) Telocytes (TC; blue arrows) positive for PDGFRα are seen in the papillary dermis of uninvolved skin. TCs have very long prolongations that run parallel to the basement membrane of the epidermis. (C) PDGFRα-positive telocytes (TC; blue arrows) are observed among the increased cellularity of the papillary dermis of treated skin and have the same orientation as in uninvolved skin. The blood vessels have comparable diameters to those in uninvolved skin. Black arrows indicate blood vessels. Magnification 200×.

**Figure 6:**
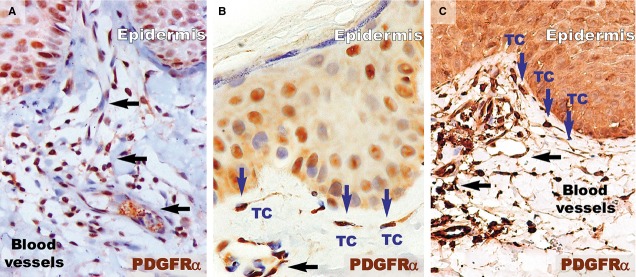
Higher magnifications of immunohistochemistry for PDGFRα in the papillary dermis show the absence of telocytes but the presence of inflammatory cells (mainly lymphocytes) in a psoriatic plaque (A). However, in the distant uninvolved skin (B), telocytes (TC, blue arrows) with very long telopodes are situated in the papillary dermis and run parallel to the line of the dermal-epidermal junction. (C) Telocytes (TC; blue arrows) were also observed in the papillary dermis of treated skin, with their telopodes running parallel to the basement membrane. Black arrows indicate blood vessels. Magnification 400×.

PDGFRα-positive cells (Fig.5) were present in the papillary dermis of distant uninvolved skin. These elongated cells with very long cellular prolongations were situated below the basement membrane and ran parallel to the basement membrane. These cells were also found close and/or enwrapping the blood vessels. The reaction for PDGFRα was increased in the papillary dermis of the lesional dermis, probably in the context of the chronic inflammation that characterizes psoriasis. However, no visible PDGFRα fusiform cells were found in the dermis. The healing process resulted in increased positivity for PDGFRα within the papillary dermis. Higher magnifications (Fig.6) revealed the presence of elongated cells in the papillary dermis, close to the basement membrane. The cellular processes were very long and moniliform. Such cells were absent in the psoriatic papillary dermis, but they were also found in treated psoriatic skin lesions, bordering the basement membrane.

**Figure 7:**
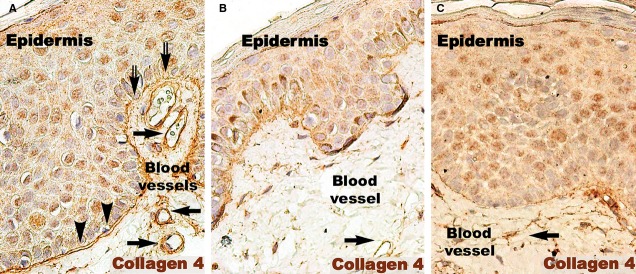
Collagen 4 immunohistochemistry of a psoriatic plaque (A), distant uninvolved skin (B), and treated skin (C). (A) Immunohistochemistry revealed the bi-layer aspect of the basal membrane in the psoriatic plaque (arrow heads). Focally, the continuity of the basal membrane was interrupted (double arrows). This aspect is different from the single line continuum of the distant uninvolved skin (B) and treated skin (C). Black arrows indicate blood vessels. Magnification 200×.

The basement membrane appeared mostly as a continuous layer in IHC for col-4, but foci of discontinuity were observed in active psoriatic lesions (Fig.7A). The basal cells of the epidermis seemed to protrude into the loose connective tissue of the papillary dermis. Moreover, the basement membrane had segments with a stratified appearance and visible space between sublayers. The space between keratinocytes in the basal and supra-basal layers seemed to be increased. As in the case of non-lesional skin, treated lesions had a continuous basement membrane (Fig.7B and C).

**Figure 8:**
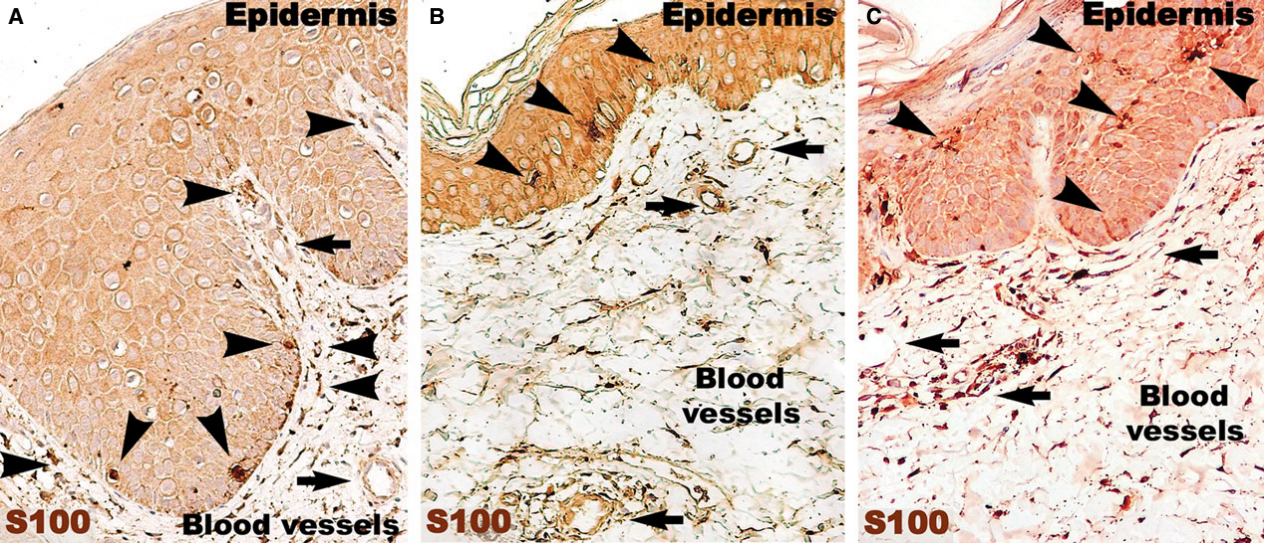
S100 immunohistochemistry of a psoriatic plaque (A) reveals an increased density of S100-positive Langerhans cells (arrow heads) beneath the basal membrane of the epidermis, and just a few positive Langerhans cells in the epidermis. In both distant uninvolved skin (B) and treated skin (C), S100-positive Langerhans cells (arrow heads) are situated in the epidermis. Black arrows indicate blood vessels. Magnification 200×.

The number of S100-positive cells (Fig.8) was not different in psoriatic skin and distant uninvolved skin. However, in the psoriatic skin, the S100-positive cells were predominantly situated in the papillary dermis among the basal cells of the epidermis. In contrast, the uninvolved skin had S100-positive DCs in the supra-basal layers of epidermis. In treated skin, S100-positive cells were also found in the entire thickness of the epidermis.

**Figure 9:**
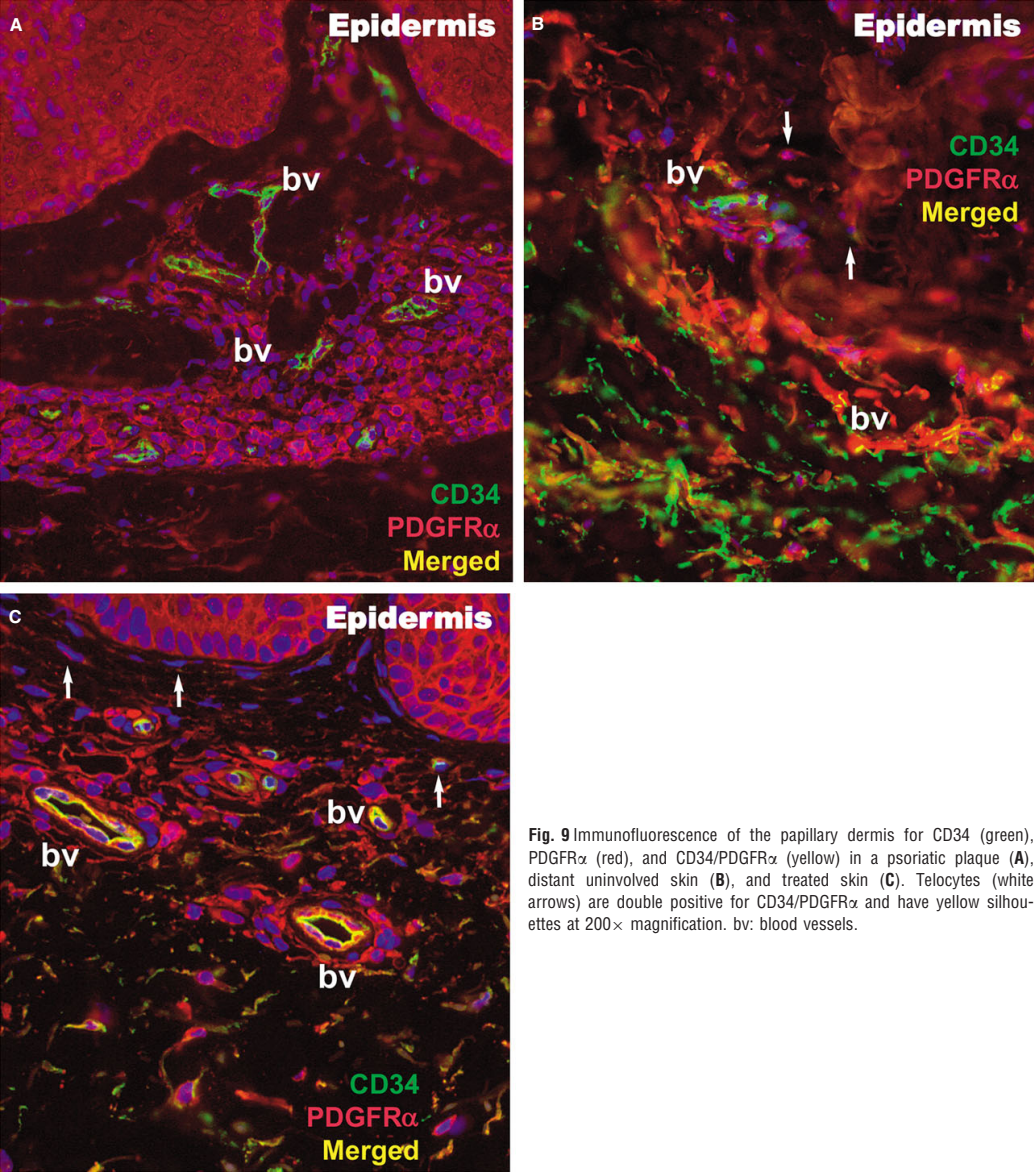
Immunofluorescence of the papillary dermis for CD34 (green), PDGFRα (red), and CD34/PDGFRα (yellow) in a psoriatic plaque (A), distant uninvolved skin (B), and treated skin (C). Telocytes (white arrows) are double positive for CD34/PDGFRα and have yellow silhouettes at 200× magnification. bv: blood vessels.

**Figure 10:**
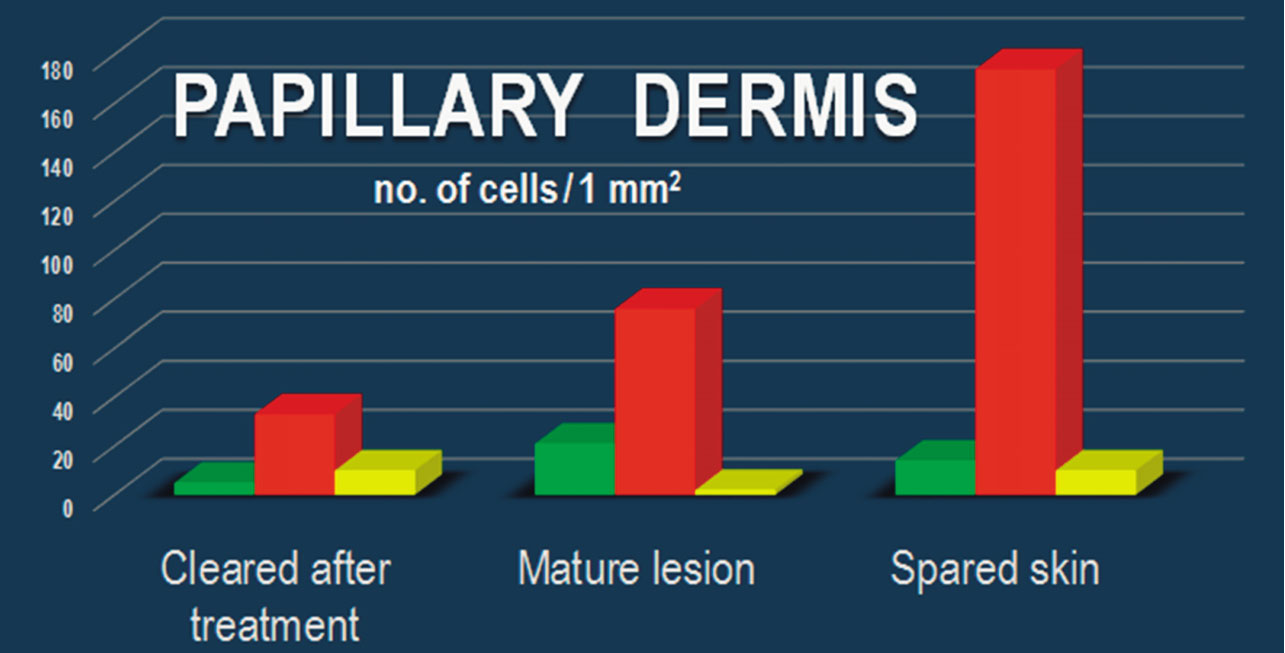
Dynamics of the number of CD34^+^ cells, PDGFRα^+^ cells, and CD34^+^/PDGFRα^+^ cells in the dermis of uninvolved skin (A), lesional skin (B), and treated skin (C).

Within the papillary and reticular dermis of uninvolved skin, IF for CD34 and PDGFRα revealed multiple double-positive cells (Fig.9). The appearance of these CD34/PDGFRα-positive cells is indicative of TCs, presenting a small cell body and very long prolongations at high magnifications. The density of these cells calculated on IF images of the papillary dermis was 10 cells/mm^2^ (Fig.10).

In the lesional reticular dermis, the measured density of CD34/PDGFRα-positive cells was comparable to that of the non-lesional reticular dermis (Fig.10), but in the lesional papillary dermis it was decreased (Fig.10). In both sublayers of the dermis, the general positivity for PDGFRα was increased in cells belonging to different structures. This could be perceived in the context of the extensive inflammatory process and substantial angiogenesis documented by IHC (Figs2 and 3). The endothelial cells positive for CD34 in dilated blood vessels were observed in both papillary and reticular dermis.

Both papillary and reticular dermis in treated skin had a considerable decrease in general positivity for PDGFRα. Interstitial cells expressing double CD34/PDGFRα positivity were also observed (Figs9 and 10). Their particular conformation with long cellular prolongations emerging from a small cell body was suggestive of TCs (Fig.9). The density of CD34/PDGFRα double-positive interstitial cells in treated skin was similar to that of uninvolved papillary dermis, but it was increased in the reticular dermis (Fig.10).

Transmission electron microscopy analyses focused on the connective tissue of the papillary dermis with an emphasis on TCs. TCs were identified based on their characteristic ultrastructural morphology as interstitial cells with long cellular processes (Fig.1A). TCs with normal morphology were frequently present in non-lesional skin (Fig.1A) but rare in the papillary and reticular dermis from psoriatic skin (Fig.1B and C). Moreover, the TCs exhibited degenerative ultrastructural features in psoriatic skin (Figs1B, C and 12). Transmission electron microscopy revealed TCs with apoptotic nuclei (Fig.1C), dystrophic TCs with fragmented Tps (Fig.1B), and TCs with nuclear extrusions and cytoplasmic disintegration (Fig.2A) in psoriatic lesions. We found no homocellular contacts between TCs in psoriatic skin. Extruded nuclei (Fig.2B) or apoptotic TCs (Fig.1C) were often observed to have close contacts with DCs in the dermis of psoriatic skin.

**Figure 11:**
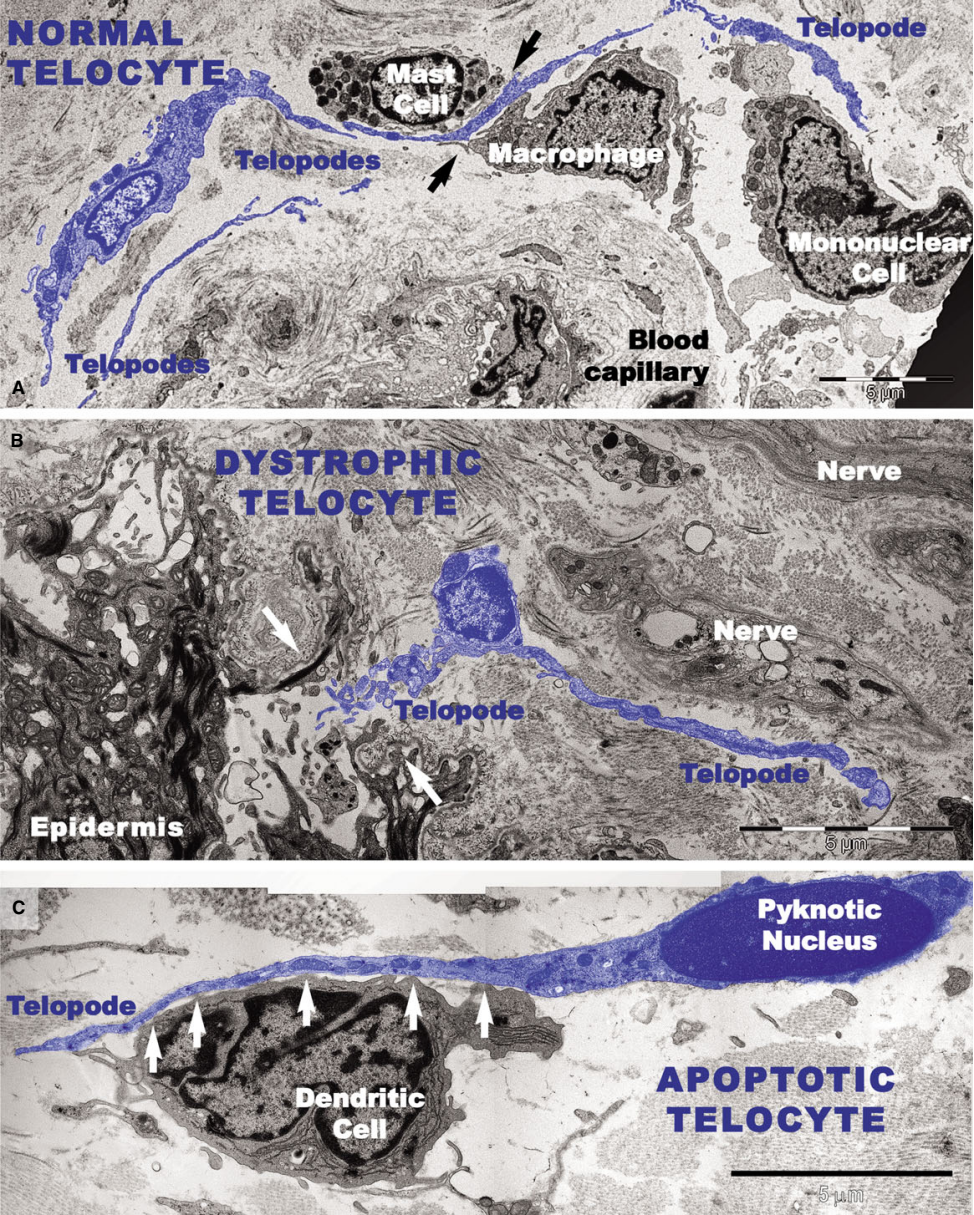
Digitally coloured transmission electron microscope images highlight telocytes in blue. (A) A telocyte with long telopode alongside a mast cell, a macrophage, and a mononuclear cell surrounding a blood vessel in uninvolved skin. Close contacts between telocytes and mast cells and between telocytes and macrophages are visible (black arrows). (B and C) TEM shows altered morphology of telocytes in the papillary dermis of a psoriatic plaque. (B) A telocyte with reduced perinuclear cytoplasm is visible beneath the epidermis at the site of a broken (white arrows) basement membrane. (C) An apoptotic telocyte with condensed chromatin in the nucleus has close contacts (white arrows) with a dendritic cell in the dermis of a psoriatic plaque. The dendritic cell has short processes.

**Figure 12:**
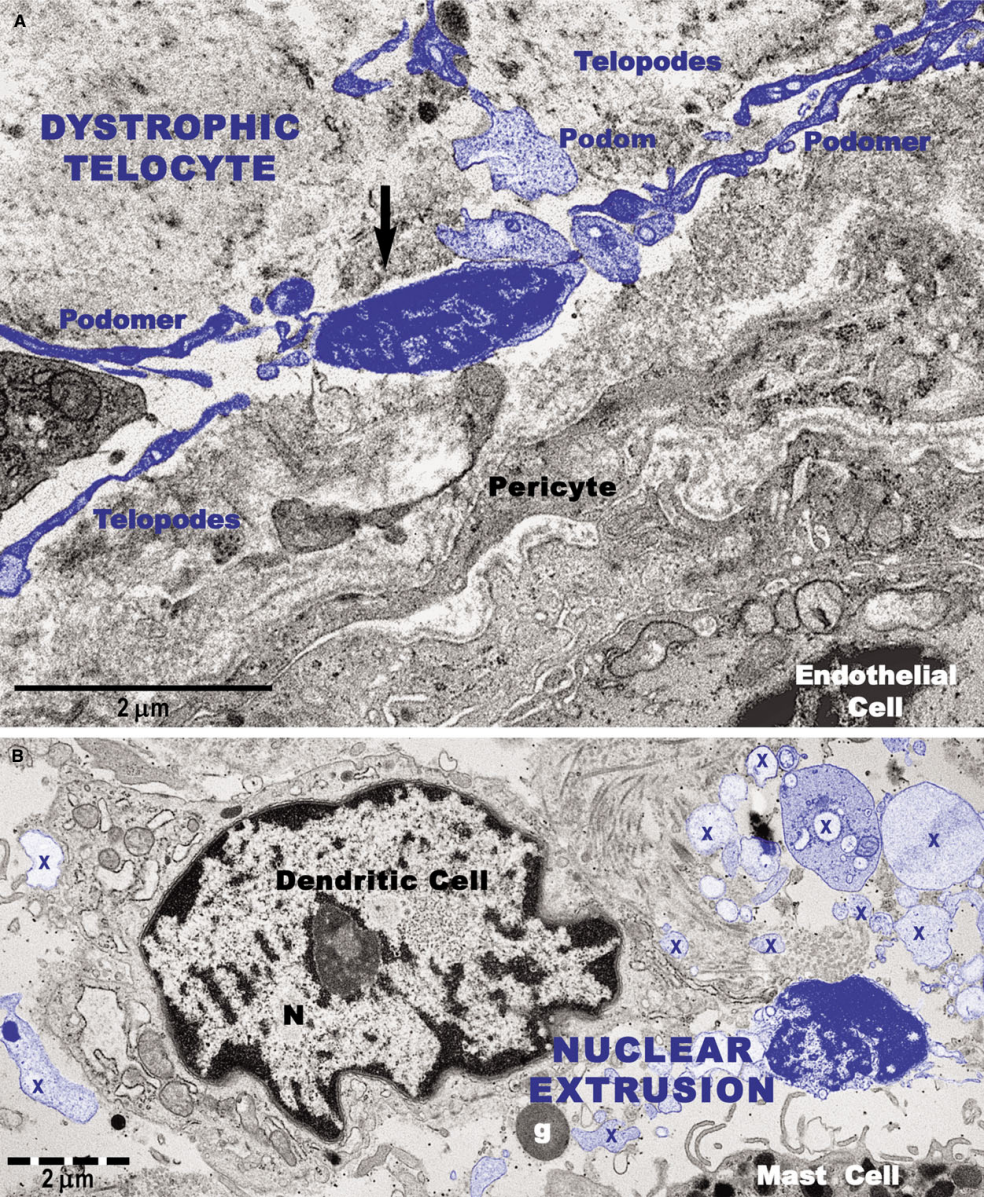
Transmission electron microscope images show degenerative changes in telocytes (digitally coloured in blue) from a psoriatic plaque. (A) A telocyte with shrivelled nucleus and detached telopodes. The arrow indicates dissolution of the cellular membrane and the cytoplasmic content surrounding the nucleus. (B) An extruded nucleus and cytoplasmic fragments (X) of a telocyte are visible in the vicinity of a dendritic cell. g: granule (of a mast cell).

DCs were frequently detected in psoriatic plaques and identified by TEM based on their morphology as cells with a stellate or tree-like appearance and numerous fine processes (Figs1C and 3). Among DCs known to be present in psoriatic lesions, LCs and pDCs were identified because of their characteristic ultrastructural features of Birbeck granules (Fig.3A and B) and abundant rough endoplasmic reticulum respectively (Fig.3C). LCs migrated from the epidermis to the dermis through the broken basement membrane (Fig.3A) and were observed in the papillary dermis (Fig.3B). The epidermal-dermal basement membrane in psoriatic plaques often showed breaks (Figs1B and 3A) with col-4 staining (Fig.7) but was continuous in non-lesional skin.

**Figure 13:**
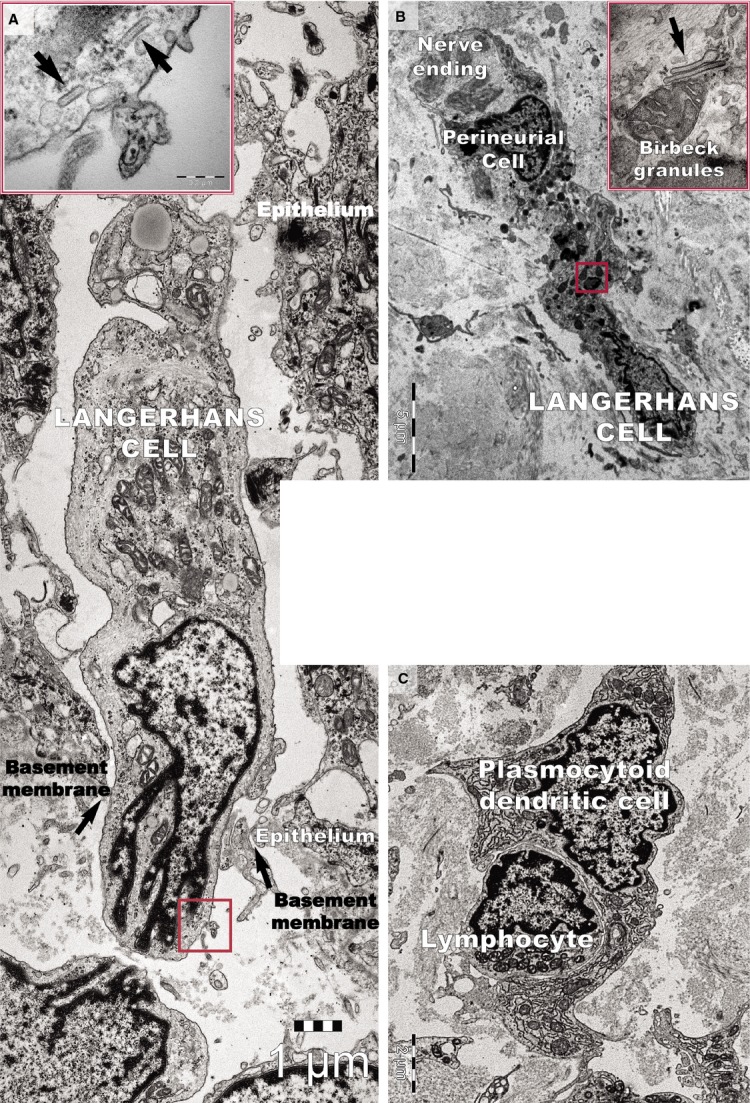
Transmission electron microscope image of a psoriatic plaque shows (A) a Langerhans cell migrating from the epidermis to the dermis through a gap (arrows) in the basement membrane. The inset shows a higher magnification of the rectangular area, revealing rod-shaped Birbeck granules (arrows). (B) A Langerhans cell from a psoriatic plaque located in the papillary dermis has a cytoplasm filled with lysosomes. The inset shows a higher magnification of the rectangular area, revealing the characteristic Birbeck granules. (C) A plasmacytoid dendritic cell surrounds a lymphocyte in the papillary dermis of a psoriatic plaque. Note the well-developed rough endoplasmic reticulum.

Ultrastructural changes were visible on vascular smooth muscle cells (VSMCs) in small blood vessels in psoriatic skin (Fig.4). In addition to the normal contractile phenotype of VSMCs (Fig.4A), the cells often exhibited a synthetic phenotype with decreased actin filaments and increased rough endoplasmic reticulum (Fig.4B and C). The VSMCs with a synthetic phenotype were hypertrophic (Fig.4B) or atrophic (Fig.4C) and had loose connections with endothelial cells (Fig.4C). Notably, the blood vessels containing VSMCs with the synthetic phenotype were not surrounded by TCs (Fig.4B and C).

**Figure 14:**
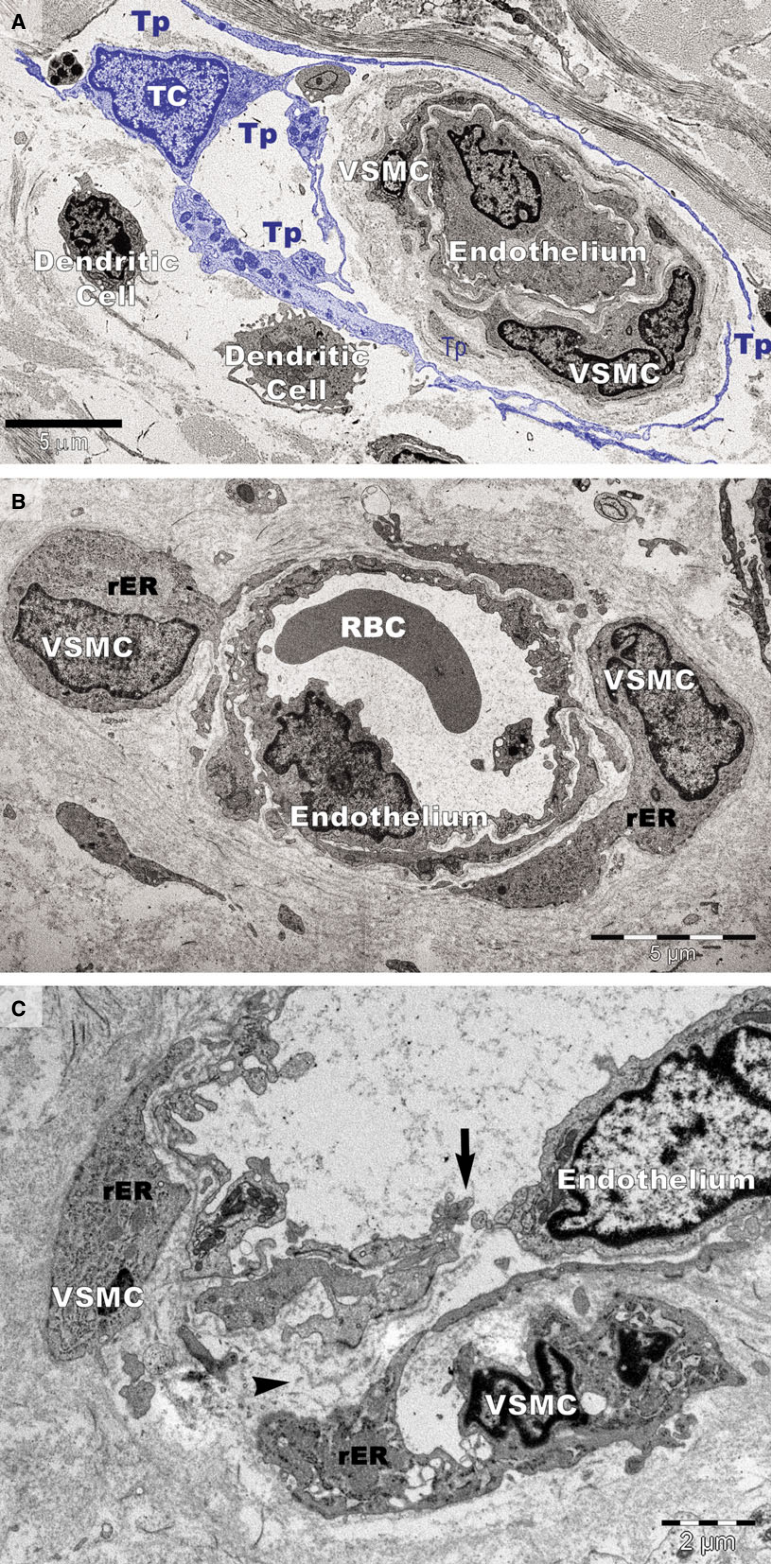
Transmission electron microscopy of small blood vessels in psoriatic plaques. (A) Vascular smooth muscle cell (VSMC) of contractile phenotype is visible in a terminal arteriole surrounded by the telopodes (Tp) of a telocyte (TC; digitally coloured in blue). Dendritic cells have short processes. (B) VSMCs with abundant rough endoplasmic reticulum (rER) in the cytoplasm are bulging into the perivascular space (hypertrophy). RBC: red blood cell. (C) VSMCs of synthetic phenotype in a terminal arteriole are detached from the endothelial layer. A gap (arrow) is visible between endothelial cells that lost their junctions. The basement membrane of the endothelium is also discontinuous (arrowhead).

## Discussion

The pathogenesis of psoriasis is not fully understood despite efforts to focus on cellular types and signalling molecules 44,55. Here, we investigated the dynamics of TCs in the vulgar form of psoriasis. As TCs are thought to be key players in the regulation of tissue/organ homoeostasis 2, our data suggest that TC loss may have important pathophysiological implications in psoriasis.

We found a decreased number of TCs in psoriatic papillary dermis and observed their recovery after local corticoid therapy. Immunofluorescence showed that the density of TCs identified as CD34/PDGFRα-positive cells was comparable in the dermis of uninvolved and treated skin but decreased in the lesional papillary dermis. Electron microscopy showed that TCs undergo apoptosis and dystrophic changes in psoriatic plaques. Also, IHC showed that CD34-positive cells are not present near dilated capillaries in the dermis of psoriatic plaques. Notably, the interstitial expression of CD34 was slightly increased in the proximity of blood vessels in treated lesional skin. These data suggest TC recovery after topic corticoid therapy despite the residual inflammatory microenvironment 44.

We found that TCs usually surround blood vessels in normal skin 2. This study shows noticeable changes in the phenotype of VSMCs in small blood vessels that are not surrounded by TCs in the papillary dermis of psoriatic skin. The tortuous, widened, elongated capillaries seem to play a central role in the pathogenesis of psoriasis 71, and endothelial cell gaps in psoriatic vessels have been reported 72, but we found no information on VSMC phenotype changes or the involvement of adventitial cells usually referred to as veil cells 72.

The 3D reconstruction of dermal TCs by FIB-SEM tomography revealed various conformations of Tps: long, flattened irregular veils (ribbon-like segments) and tubular structures (podomers) with uneven calibre because of irregular dilations (podoms) 26. This 3D appearance of TCs is similar to that of ‘veil cells’ described in the skin 72. The exact nature and function of these cells are still undetermined considering they lack cell markers for T, B, LCs, or HLA-DR 72.

Vasodilatation tests indicated that arterioles in psoriatic plaques are not normally maximally dilated but have a basal constrictor tone 73. Loss of the contractile phenotype of VSMCs in arterioles from plaques could cause the phenotype change in VSMCs and, consequently, the structural widening of arterioles. Studies have shown that phenotype alterations and differentiation of VSMCs are important for angiogenesis, blood vessel remodelling and homeostasis, and both the composition and organization of the extracellular matrix (ECM) have major consequences for the smooth muscle cell phenotype 74,75. Preferential distribution of TCs around blood vessels could be important in vascular physiology, and TCs certainly contribute to the composition and organization of the ECM. The loss of perivascular TCs could trigger the characteristic vascular pathology in psoriasis. Moreover, the loss of TCs could be considered a mechanism underlying the most pathognomonic sign of psoriasis vulgaris, Auspitz’s sign.

We previously showed that, within the dermis, TCs form an interstitial network based on homocellular (TC-TC junctions) and heterocellular (TC-other interstitial cells) interactions, suggesting an essential role of TCs in intercellular signalling required for skin homeostasis 2. Recent studies have shown that TC injury may have important pathophysiological implications in systemic sclerosis 36,37. The loss of TCs and their interstitial network may significantly influence psoriatic lesion initiation and/or pathology progression, impairing long distance heterocellular communication.

In conclusion, damaged TCs could be an important step in the pathophysiology of psoriasis. This study offers new insights into the cellularity of psoriatic lesions and we suggest considering TCs as new cellular targets for forthcoming therapies. Because pustular psoriasis is not characterized by Auspitz’s sign, clarifying the involvement of TCs in other forms of psoriasis would be attractive. In the future, we propose extending the research to the pustular and erythrodermic forms of psoriasis.
